# Herbal medicines for the treatment of otitis media with effusion: a systematic review of randomised controlled trials

**DOI:** 10.1136/bmjopen-2016-011250

**Published:** 2016-11-24

**Authors:** Mi Ju Son, Songie Choi, Young-Eun Kim, Yun Hee Kim

**Affiliations:** 1 Clinical Research Division, Korea Institute of Oriental Medicine, Daejeon, Republic of Korea; 2 K-herb Research Center, Korea Institute of Oriental Medicine, Daejeon, Republic of Korea; 3 Mibyeong Research Center, Korea Institute of Oriental Medicine, Daejeon, Republic of Korea; 4 KM Convergence Research Division, Korea Institute of Oriental Medicine, Daejeon, Republic of Korea

**Keywords:** Otitis Media with Effusion, Systematic Review

## Abstract

**Objectives:**

This systematic review aimed to assess the clinical evidence supporting the use of herbal medicines (HMs) for the treatment of otitis media with effusion (OME).

**Design:**

Systematic review and meta-analysis.

**Data sources:**

MEDLINE, EMBASE, Cochrane Library, AMED, CINAHL and three trial registries were searched up to January 2015. We also searched five Korean medical databases (KoreaMed, RISS, OASIS, DBPIA and KISS) and three Chinese databases (CNKI, Wanfang and VIP).

**Study eligibility criteria:**

This study included randomised clinical trials that reported the effects of HM for OME. The primary outcome was the complete resolution of OME at 2 or 3 months post randomisation. Secondary outcomes included the partial or complete resolution at all possible time points and hearing test. Three authors independently screened the titles and abstracts, selected studies and extracted the data relating to trial quality, characteristics and results.

**Results:**

A total of 2141 potentially relevant studies were identified, of which 17 randomised clinical trials met our inclusion criteria. Most were evaluated as having a high or unclear risk of bias. Tongqiao tablets, Tongqiao huoxue decoctions and Tsumura-Saireito were associated with a lower complete or partial resolution rate when compared with conventional medicines (CMs) (p=0.02, p=0.0001, and p=0.04, respectively), and similar outcomes were observed with Huanglong tonger pills, Erzhang decoctions and Shenling baizhu powder when combined with CM versus CM alone (p<0.00001, p=0.02, and p=0.05, respectively). Tongqiao huoxue decoction plus CM appeared to be more effective than CM in terms of improving the pure tone threshold levels (p=0.0007). Tsumura-Saireito was found to affect the proportion of patients with normalised tympanometry (p=0.03).

**Conclusions:**

Despite some indications of potential symptom improvement, the evidence regarding the effectiveness and efficacy of HMs for OME is of poor quality and therefore inconclusive.

**Protocol registration number:**

CRD42013005430.

**Strengths and limitations of this study**

- This is the first systematic review to provide an evidence of the use of herbal medicine (HM) for the treatment of otitis media with effusion (OME).
- Our systematic review involved an unbiased search of various databases without language restriction.
- Our systematic review will give readers the opportunity to access studies originally published in East Asian languages that they would otherwise be unable to read.
- Despite some indications of potential improvement of symptoms, the evidence regarding the effectiveness and efficacy of HMs for OME is of poor quality and therefore inconclusive.

## Introduction

Otitis media with effusion (OME) is characterised by middle ear effusion without symptoms and signs of acute inflammation.^1^ OME occurs commonly during childhood, affecting 50–90% of children at least once by 5 years of age.^2^ ^3^ At least 25% of OME episodes persist for more than 3 months, and may be associated with hearing loss, balance problems, poor school performance, behavioural problems, ear discomfort, recurrent acute otitis media and reduced quality of life.^4–7^

The most widely used therapeutic agents for OME attempt to mitigate symptoms and eliminate effusion. The therapeutic agents include antihistamines, decongestants, steroids and antibiotics; however, antihistamines, decongestants and topical nasal steroids are known to be ineffective.^8–10^ Antibiotics are not recommendable because their adverse effects outweigh their small benefit.^11^ Short-term treatment with oral steroids is effective, but extending the treatment for longer than 2 weeks has no added benefit.^12^ The most recommended treatment is ventilation tube insertion, which is considered when OME persists after a 3-month period of watchful waiting.^13^

Oral administration of herbal medicines (HMs) has been broadly used to manage OME in East Asian countries.^14^ According to animal experiments involving herbal preparations, a Ginkgo leaf parenteral solution conferred protection against oxidative injuries in rats with otitis media by increasing antioxidant and immune activity,^15^ whereas the HM Saireito induced much milder pathological changes in the tubotympanum and stimulated ciliary activity in guinea pigs.^16^ Additionally, the HM, Kami-hyunggyeyungyotang was found to induce antiallergic and antioxidant effects by regulating the production of immunoglobulin G, interleukin-8, cytokines, tryptase, superoxide dismutase and transforming growth factor-β1 in patients with recurrent OME.^17–20^

Several randomised controlled trials (RCTs) have evaluated the effects of HM on OME. Unfortunately, these studies have reached conflicting conclusions, with only some demonstrating the clinical benefits of HM as a main or adjunct treatment. In this review, we aimed to systematically accumulate evidence regarding the safety and effectiveness of HM for patients with OME.

## Methods

### Protocol and registration

This systematic review was registered in an international prospective register of systematic reviews under the registration number PROSPERO 2013: CRD42013005430 (available from: http://www.crd.york.ac.uk/prospero/display_record.asp?ID=CRD42013005430#.VWcfac_tlBc).

### Data sources and searches

The following electronic databases were searched up to January 2015: MEDLINE, EMBASE, Cochrane Central Register of Controlled Trials, AMED and the Cumulative Index to Nursing and Allied Health Literature. We also searched five Korean medical databases (KoreaMed, RISS, OASIS, DBPIA and KISS) and three Chinese databases (CNKI, Wanfang and VIP). Additionally, we sought on-going studies in the meta-Register of Controlled Trials (http://www.controlled-trials.com/mrct), Clinical trials.gov (http://www.clinicaltrials.gov) and the WHO International Clinical Trials Registry platform (http://apps.who.int/trialsearch/), all of which list on-going trials. No limits or filters were placed on the searches to ensure maximal sensitivity, and no language or publication type restrictions were applied. The MEDLINE database search strategy is presented in online supplementary appendix 1. Similar search strategies were applied for the other databases.

10.1136/bmjopen-2016-011250.supp1 supplementary appendix 1

### Study selection

Study selection was based on the following criteria:

*Type of study:* RCTs that reported the effects of HM on OME were included. Trials that did not provide detailed information (eg, dosage or comparison data) were excluded.

*Type of participant:* Studies that evaluated patients with a diagnosis of OME were included. We excluded studies of patients in whom ventilation tubes had been placed, those with an anatomical deformity or those with other chronic immunocompromised states.

*Type of intervention:* We included trials that evaluated orally administered HM alone or in combination with a conventional medicine (CM) versus CM alone. We included all types of herbal formulations. Trials that incorporated herbal decoctions but did not provide detailed information such as the herbal dosage, preparation, or HM addition and subtraction criteria were excluded. However, we included trials using HMs manufactured by pharmaceutical companies, regardless of sufficient herbal prescription and dosage information.

*Type of comparison:* Both active control and placebo were acceptable.

*Types of outcome measures:* The primary outcome was the complete resolution of OME at 2 or 3 months post randomisation (resolution in the affected ear in participants with unilateral OME at randomisation and resolution in both ears of those with bilateral OME). We also planned to evaluate the partial or complete resolution of OME at all possible time points, hearing loss duration, language and speech development, cognitive development, ventilation tube insertion, tympanic membrane sequelae, reduction in OME complications, quality of life and adverse effects likely related to treatment. All studies including any of the above outcome measures were evaluated.

### Data extraction and quality assessment

Two authors (MJS, Y-EK) independently screened the titles and abstracts, selected the studies and extracted the data from studies in international and Korean databases using a standard eligibility form; two other authors (MJS, SC) performed the same tasks in Chinese databases.

Data concerning the patient population characteristics, HM treatment regimens and comparators, reported outcomes and assessment modality (if reported) were collected from each trial. The arbitrator (YHK) made decisions regarding study selection and extraction when a consensus could not be reached. The risk of bias in the eligible studies was independently assessed according to the criteria described in the Cochrane Handbook V.5.1.0.^21^ The quality of each study was classified as a low, unclear or high risk of bias. Any differences in opinion were resolved via discussion or arbitration involving a third author.

### Statistical analysis

We used RevMan 5.3.5 (Cochrane Informatics and Knowledge Management Department; available at http://tech.cochrane.org/revman/download) to conduct the statistical analysis. Dichotomous data were expressed as risk ratios (RRs) with 95% confidence intervals (CIs), whereas continuous data were presented as mean differences (MDs) with 95% CIs. We converted other forms of data into either RRs or MDs. The level of significance was set at 0.05. Heterogeneity was assessed using the I^2^ statistic to quantify inconsistency among the included studies in the meta-analysis.
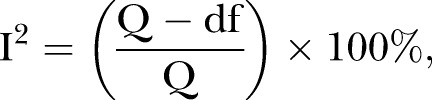


where Q is the χ^2^ statistic and df is its degrees of freedom.

An I^2^ value >50% was considered indicative of substantial heterogeneity according to Cochrane guidelines.^21^ A Z-test was used for testing overall effects in the meta-analysis. Funnel plots were planned to detect publication bias if more than 10 trials reported the same outcomes. If data were available, a predefined subgroup analysis was planned to evaluate heterogeneity. The predefined subgroup analysis was planned to evaluate the following information: (1) Laterality of OME: bilateral OME versus unilateral OME, (2) Duration of OME: any duration of OME versus persistent OME (lasting for more than 2 or 3 months), (3) Duration of treatment, (4) Type of HMs, (5) Type of control and (6) Type of age group.

If the included studies were sufficient to perform a sensitivity analysis, analyses according to sample size (>40 or <40 participants) and risk of bias (low risk of bias in allocation concealment or the blinding participants/assessors) were planned.

## Results

### Study selection and description

Our search generated a total of 2141 potentially relevant studies, from which 80 duplicated and 1963 non-relevant studies were excluded after screening the titles and abstracts. Subsequently, 98 full-text articles were reviewed, of which 17 met our eligibility criteria. Five RCTs did not meet our inclusion criteria; four trials included patients who inserted ventilation tubes and one trial evaluated combination therapy of HM plus microwaves.

**Figure 1:**
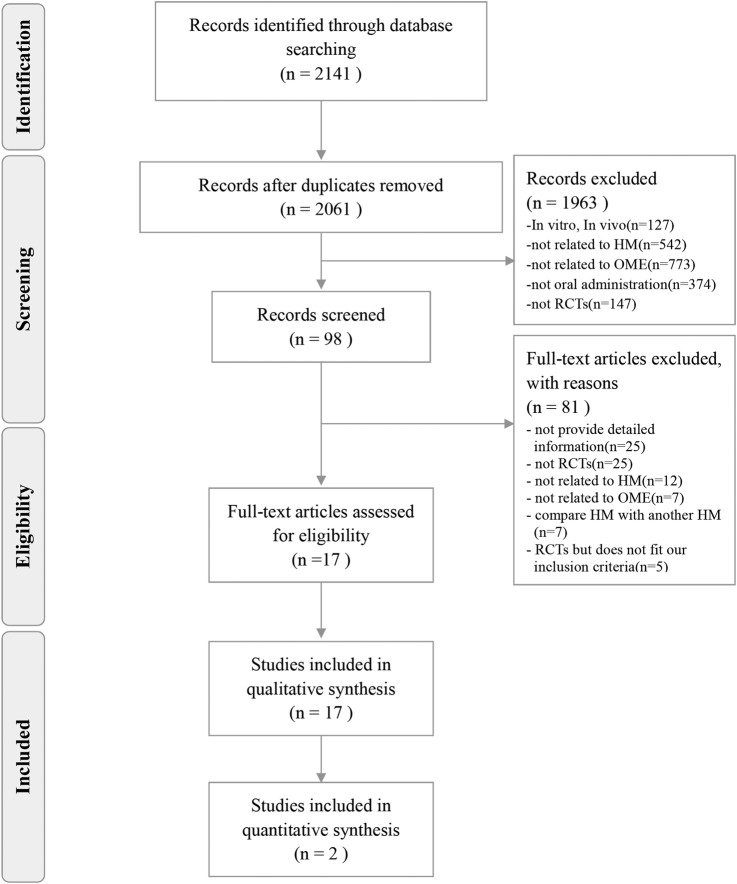
PRISMA flow diagram.

The Preferred Reporting Items for Systematic Reviews and Meta-Analyses (PRISMA) flow diagram of our search process and study selection is shown in figure 1.

Of these trials, 16^22–37^ were conducted in China; of these, 15^22–33^ ^35–37^ were published in Chinese and 1^34^ was published in English. One additional trial^38^ was conducted in Japan and published in Japanese. In total, 3161 participants with OME were involved in the 17 trials. Fifteen trials^22–30^ ^32–34^ ^36–38^ involved participants younger than 18 years, and two^36^ ^38^ involved only children under 7 years of age.

Seven trials^24–26^ ^28^ ^30^ ^34^ ^38^ compared HM treatments with CMs; the remaining trials compared combination treatments involving HM and CM with CM alone. Two trials^31^ ^34^ reported data concerning the complete resolution of clinical symptoms and signs at 2 or 3 months post randomisation. All included trials reported either complete or partial resolution at various time points. Only five trials^28–30^ ^34^ ^38^ reported information about adverse events and none of the trials mentioned ethical issues.

Key data points from the included RCTs are summarised in table 1.

**Table 1:** Basic characteristics of the included studies

First author (year), country	Mean age (range)	Duration of disease (range)	Sample size (male/female)	Unilateral/bilateral	Experimental intervention (regimen)	Control intervention (regimen)	Outcome measure	Adverse effects
Chen (2013), China^22^	(A) 39.7±6.0 (6–60) (B) 37.2±6.2 (7–58)	(A) 31.2±9.2 months (8–42 weeks) (B) 29.8±9.6 months (8–39 weeks)	(A) 30 (16/14) (B) 30 (13/17)	(A) 17/13 (B) 21/9	(A) HM (Tongqiao huoxue decoction) 150 mL once a day for 14 days plus (B)	(B) 1% Ephedrine hydrochloride and nitrofurazone nasal drops 2 times a day, roxithromycin 150 mg 2 times a day plus prednisone 30 mg once a day for 14 days (PRN) Glucocorticoid and chymotrypsin injection	Clinical symptoms evaluation	n.r.
Guo (2004), China^23^	(A) 38.2 (6–54) (B) 42.4 (8–67)	(A) n.r. (1 week–2 years) (B) n.r. (1 week–2 years)	(A) 53 (26/27) (B) 42 (20/22)	(A) 44/9 (B) 36/6	(A) HM (Biyan Qingdu granule) 20 g each time 2 times a day plus (B) for 2 weeks	(B) Ambroxol hydrochloride 30 mg each time 3 times a day for 2 weeks	Clinical symptoms evaluation	n.r.
He (2013), China^24^	(A) n.r (10–72) (B) n.r (9–70)	(A) n.r. (B) n.r.	(A) 55 (27/28) (B) 55 (38/17)	(A) 46/9 (B) 43/12	(A) HM (Tongqiao huoxue decoction) 500 mL divided into 3, 3 times a day for 14 days (half dose for those younger than 14 years)	(B) Roxithromycin 0.15 g 2 times a day (5 mg/kg for kids) plus prednisolone acetate 20 mg once a day for 14 days	Clinical symptoms evaluation	n.r.
Hu (2000), China^25^	(A) 45 (19–61) (B) 38 (16–68) (C) 43 (16–70)	(A) n.r. (6 months–15 years) (B) n.r. (3 months–12 years) (C) n.r. (4 months–15 years)	(A) 40 (22/18) (B) 34 (14/20) (C) 36 (14/22)	(A) 27/13 (B) 25/9 (C) 26/10	(A) HM 2 times a day for 2–4 weeks (C) (A) plus (B)	(B) Ear inflation treatment once per 3 days for 15 days plus chymotrypsin 1 mg injection once a week for 2–4 weeks	Clinical symptoms evaluation	n.r.
Jiang (2013), China^26^	(A) 35.15±12.7 (12–50) (B) 33.49±11.8 (12–50)	(A) n.r. (>8 weeks) (B) n.r. (>8 weeks)	(A) 30 (17/13) (B) 30 (15/15)	(A) 14/16 (B) 12/18	(A) HM (Shenling baizhu powder) 9 g 3 times a day for 15 days	(B) Cefetamet pivoxil hydrochloride dispersible tablets 500 mg 2 times a day plus ambroxol hydrochloride tablets 60 mg 3 times a day for 15 days	Clinical symptoms evaluation	n.r.
Li (2014), China^27^	(A) 32.2±1.4 (8–56) (B) 33.2±1.3 (8–57)	(A) 5.6±2.1 months (5 days–9 months) (B) 5.3±2.2 months (5 days–9 months)	(A) 60 (34/26) (B) 60 (29/31)	(A) n.r./n.r. (B) n.r./n.r.	(A) HM 100 mL 2 times a day plus (B) for 14 days	(B) Roxithromycin 150 mg 2 times a day plus prednisone 10 mg 3 times a day plus triamcinolone acetonide 20 mg injection for 14 days	Clinical symptoms evaluation	n.r.
Liao (1998), China^28^	(A) 35.7 (6–59) (B) 32.5 (6–59)	(A) 4.3 months (2 weeks–2 years) (B) 4.2 months (2 weeks–2 years)	(A) 52 (24/28) (B) 44 (20/24)	(A) 40/12 (B) 38/6	(A) HM (Tongqiao tablets) 2 g 3 times a day for 4 weeks	(B) Amoxicillin 0.25 g 4 times a day plus terfenadine 60 mg, 2 times a day for 4 weeks	Clinical symptoms evaluation, AE	none
Liu (2005), China^29^	(A) 37.40±11.73 (15–60) (B) 38.85±11.33 (25–65)	(A) 14.40±6.57 days (B) 18.10±6.14 days	(A) 20 (14/6) (B) 20 (15/5)	(A) 13/7 (B) 15/5	(A) HM 2 times a day plus (B) for 14 days	(B) 1% ephedrine hydrochloride nasal drops, 2 drops 3 times a day plus cefradine 0.5 g/kg 4 times a day for 14 days	Clinical symptoms evaluation, AE	none
Liu (2014), China^30^	(A) 37.2±8.3 (12–62) (B) 36.9±8.1 (11–60)	(A) n.r. (2–40 days) (B) n.r. (3–37 days)	(A) 54 (28/26) (B) 54 (29/25)	(A) 46/8 (B) 43/11	(A) HM 2 times a day for 21 days	(B) Cephradine 0.75 mg 3 times a day, plus prednisone 5 mg and mucosolvan 10 mL 2 times a day for 21 days	Clinical symptoms evaluation, AE	(A) Headache and dizziness (1) (B) Headache and dizziness (2), nausea and vomiting (2), xerostomia (1)
Lu, (2013) China^31^	(A) n.r. (18–60) (B) n.r. (18–60)	(A) n.r. (2–24 weeks) (B) n.r, (2–24 weeks)	(A) 30 (B) 30	(A) 20/10 (B) 23/7	(A) HM 150 mL 2 times a day for 14 days plus (B)	(B) Cephradine 0.25 g 3 times a day plus mucosolvan 30 mL 3 times a day for 14 days (PRN) chymotrypsin 4000 U and prednisolone acetate injection 0.5 mL once a week	Clinical symptoms evaluation	n.r.
Qu (2013), China^32^	(A) 30.3 (16–70) (B) 33.7 (18–72)	(A) 2.9 months (7 days–14 weeks) (B) 2.5 months (2 days–12 weeks)	(A) 85 (51/34) (B) 85 (53/32)	(A) 71/14 (B) 74/11	(A) HM 150 mL 3 times a day plus (B) for 14 days	(B) Roxithromycin 150 mg 2 times a day for 14 days plus triamcinolone acetonide 40 mg plus chymotrypsin 4000 U injection once a week	Clinical symptoms evaluation	n.r.
Sato (1988), Japan^38^	(A) 5.2±0.9 (4–7) (B) 5.0±0.9 (4–7)	(A) 7.7±6.2 months (0–24 months) (B) 8.9±7.4 months (0–24 months)	(A) 21 (12/9) (B) 21 (16/5)	(A) 10/11 (B) 10/11	(A) HM (Tsumura-Saireito) 1.5 g 2 times a day for 4 weeks	(B) Cepharanthin 5–7.5 mg 2 times a day for 4 weeks	Clinical symptoms evaluation, pure tone audiometry, tympanometry, AE	none
Shi (2005), China^33^	(A) 30.13 (6–71) (B) 29.51 (7–69)	(A) n.r. (1 day–18 years) (B) n.r. (1 day–16 years)	(A) 860 (540/320) (B) 810 (520/290)	(A) 770/90 (B) 730/80	(A) HM (Huanglong tonger pill) 10 g 2 times a day plus (B)	(B) Roxithromycin 150 mg 2 times a day and prednisone 10 mg 3 times a day for 3 days plus 1% ephedrine hydrochloride and nitrofurazone nasal drops 3 times a day plus tympanic inflation once per 2 days plus auripuncture	Clinical symptoms evaluation	n.r.
Sun (2005), China^34^	(A) 34.4±14.6 (5.9–68) (B) 27.9±17.0 (4–64)	(A) 81.96±124.64 days (1–730 days) (B) 130.12±157.25 days (1–730 days)	(A) 45 (23/22) (B) 45 (26/19)	(A) n.r./n.r. (B) n.r./n.r.	(A) HM (Qingqiao capsule) 5 capsules 3 times a day for 10–14 days(adjust dosage according to age)	(B) Cefaclor capsule 0.5 g for adults per each time (20 mg/kg per day for child), 3 times a day for 10–14 days	Clinical symptoms evaluation, pure tone audiometry, AE	(A) No AE (B) Nausea, vomiting and diarrhoea (1), urticaria (1)
Tian (2014), China^35^	(A) 42.48±11.90 (21–65) (B) 43.21±12.21 (22–67)	(A) 7.71±2.59 months (3–12 months) (B) 7.73±2.38 months (4–12 months)	(A) 34 (20/14) (B) 33 (17/16)	(A) 26/8 (B) 24/9	(A) HM (Shenling baizhu powder) 9 g 3 times a day for 21 days plus (B)	(B) Povidone iodine disinfection, plus 2% tetracaine 1 mL injection, plus mucosolvan 15 mg and dexamethasone 5 mg injection for 21 days	Clinical symptoms evaluation	n.r.
Zhang (2013), China^36^	(A) n.r. (1–5) (B) n.r. (1–5)	(A) n.r. (≤2 weeks) (B) n.r. (≤2 weeks)	(A) 32 (B) 31	(A) 24/8 (B) 23/8	(A) HM (Erzhang decoction) 150 mL 2 times a day plus (B) for 7 days	(B) Clarithromycin 0.25 g 2 times a day plus chymotrypsin 4000 U plus triamcinolone acetonide injection once a week plus for 7 days	Clinical symptoms evaluation	n.r.
Zhao (2012), China^37^	(A) 30.67 (7–70) (B) 30.34 (8–68)	(A) n.r. (1 day–16 years) (B) n.r. (1 day–15 years)	(A) 100 (55/45) (B) 100 (58/42)	(A) 31/69 (B) 37/63	(A) HM (Huanglong tonger pill) 2 times a day plus (B) for 10 days	(B) Roxithromycin 150 mg 2 times a day for 10 days plus prednisone 10 mg 3 times a day for 10 days plus tympanic inflation once per 2 days plus auripuncture	Clinical symptoms evaluation	n.r.

AE, adverse event; HM, herbal medicine; n.r., not reported; PRN, pro re nata.

(A), Experimental intervention; (B), Control intervention; (C), Combination of experimental and control intervention.

### Intervention

Five types of HM prescriptions were evaluated in the included trials, including herbal granules manufactured by pharmaceutical companies (four trials), practitioner-prescribed herbal decoctions (nine trials), pills (two trials), tablets (one trial) and capsules (one trial). No trials described the quality standards of the herbal preparations. Among our included trials, two investigated several different herbal prescriptions that had been prescribed individually. In a majority of the included studies, a 2-week treatment course was provided (range: 1–4 weeks). Shenling baizhu powder, Huanglong tonger pills and a Tongqiao huoxue decoction were each investigated in more than two studies. Details regarding the HM regimens used in the included trials are shown in online supplementary appendices 2 and 3.

10.1136/bmjopen-2016-011250.supp2 supplementary appendix 2

10.1136/bmjopen-2016-011250.supp3 supplementary appendix 3

Oral antibiotics were mainly used as control interventions; other conventional treatments included oral antihistamines, prednisone and mucolytic or secretolytic agents that were provided to either the control group alone or to both groups.

### Risk of bias in the included studies

We attempted to contact the authors of all included trials for clarification and details. However, few trial reports provided contact details and no authors could be contacted, despite contact attempts in Chinese via email.

Overall, the studies were found to have a high risk of bias. Most studies featured a randomised design but provided inadequate descriptions of randomisation, allocation concealment and outcome assessor blinding. Only one trial^34^ reported the use of a random number table for random sequence generation, whereas the remaining trials^22–33^ ^35–38^ did not report any randomisation details. None of the trials described their allocation concealment method. Furthermore, only one trial^34^ incorporated a double-blind design; the remaining studies^22–33^ ^35–38^ did not appear to implement both patient and practitioner blinding. None of the trials reported details regarding outcome assessor blinding.

**Figure 2:**
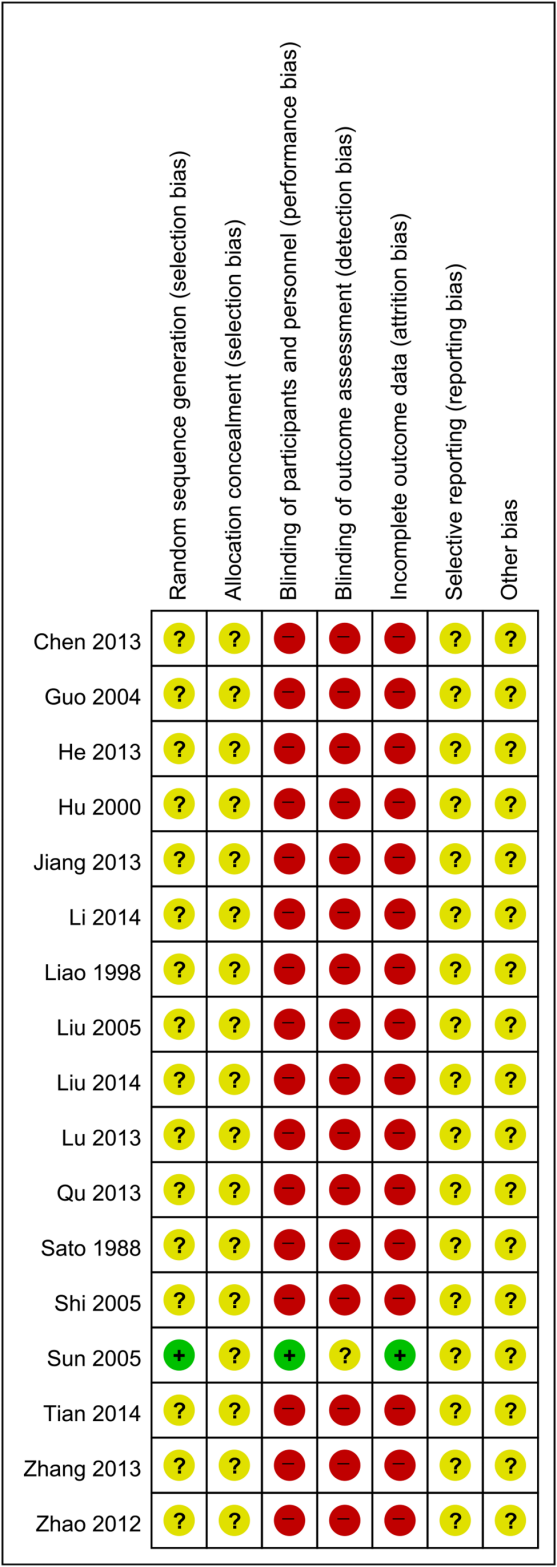
Risk of bias in the included randomised controlled trials.

Not all trials provided complete outcome data (eg, numbers of participants who were included, finished treatment and dropped out). Selective outcome reporting was not clearly evaluable because we could not find registered protocols for all included studies. In addition, not all trials reported statistical issues such as baseline imbalances and sample size calculations to ensure sufficient statistical power. Only one trial^34^ reported conducting an intention-to-treat or per-protocol analysis as an effectiveness evaluation. The risk of bias assessment is shown in figure 2.

### Outcomes

**Figure 3:**
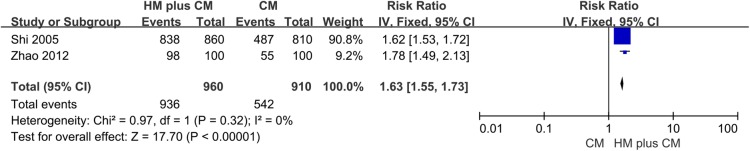
Forest plot of the complete resolution rate achieved with the Huanglong tonger pill.

Given the heterogeneity of the HM treatment and control groups in the included trials, we could only synthesise data from two trials into a meta-analysis. The effect estimates of the included trials are shown in table 2, and meta-analysis results are indicated in figure 3.

**Table 2:** Estimated effects of herbal medicine on improvements in the clinical outcomes of patients with otitis media with effusion

Outcomes	No. of studies	Effect estimates	p Value	Study
Proportion of patients with complete resolution (outcome evaluation date ≤1 week)				
Huanglong tonger pill plus CM vs CM	1	RR 2.12 (1.94 to 2.31)	<0.00001	Shi *et al*^33^
Erzhang decoction plus CM vs CM	1	RR 1.65 (1.07 to 2.54)	0.02	Zhang *et al*^36^
Proportion of patients with complete resolution (outcome evaluation date >1 week, ≤2 weeks)				
Biyan Qingdu granule plus CM vs CM	1	RR 1.29 (0.88 to 1.90)	0.20	Guo *et al*^23^
HM plus CM vs CM	1	RR 3.15 (1.37 to 7.26)	0.007	Liu *et al*^29^
Huanglong tonger pill plus CM vs CM	2	RR 1.63 (1.55 to 1.73)	<0.00001	Shi *et al*^33^, Zhao *et al*^37^
Tongqiao huoxue decoction plus CM vs CM	1	RR 1.68 (0.75 to 3.79)	0.21	Chen *et al*^22^
HM plus CM vs CM	1	RR 1.08 (0.58 to 2.02)	0.81	Lu *et al*^31^
HM plus CM vs CM	1	RR 1.37 (0.93 to 2.02)	0.11	Qu *et al*^32^
HM plus CM vs CM	1	RR 1.37 (0.97 to 1.93)	0.07	Li *et al*^27^
Proportion of patients with complete resolution (outcome evaluation date >2 weeks, ≤4 weeks)				
Tongqiao tablets vs CM	1	RR 3.05 (1.23 to 7.54)	0.02	Liao *et al*^28^
HM vs CM	1	RR 1.06 (0.31 to 3.65)	0.92	Hu *et al*^25^
Shenling baizhu powder vs CM	1	RR 1.91 (0.77 to 4.75)	0.16	Jiang *et al*^26^
HM vs CM	1	RR 1.43 (1.11 to 1.84)	0.006	Liu *et al*^30^
HM plus CM vs CM	1	RR 2.83 (1.01 to 7.94)	0.05	Hu *et al*^25^
Shenling baizhu powder plus CM vs CM	1	RR 1.55 (1.01 to 2.39)	0.05	Tian *et al*^35^
Proportion of patients with complete resolution (outcome evaluation date >4 weeks, ≤8 weeks)				
Tongqiao huoxue decoction vs CM	1	RR 3.35 (1.80 to 6.24)	0.0001	He *et al*^24^
Proportion of patients with complete resolution (outcome evaluation date >8 weeks)				
Qingqiao capsule vs CM	1	RR 2.33 (0.98 to 5.53)	0.05	Sun *et al*^34^
HM plus CM vs CM	1	RR 1.01 (0.51 to 2.00)	0.98	Lu *et al*^31^
Proportion of patients with partial resolution (outcome evaluation date =4 weeks)				
Tsumura-Saireito vs CM	1	RR 2.33 (1.03 to 5.30)	0.04	Sato *et al*^38^
Score of pure tone audiometry (dB)				
Tsumura-Saireito vs CM	1	MD 3.30 (−1.88 to 8.48)	0.21	Sato *et al*^38^
Tongqiao huoxue decoction plus CM vs CM	1	MD 5.80 (2.44 to 9.16)	0.0007	Chen *et al*^22^
Evaluation restoration rate of pure tone audiometry				
Qingqiao capsule vs CM	1	RR 1.61 (1.12 to 2.32)	0.010	Sun *et al*^34^
Evaluation restoration time of pure tone audiometry				
Qingqiao capsule vs CM	1	MD −1.70 (−2.50 to −0.90)	<0.0001	Sun *et al*^34^
Proportion of patients with hearing improvement				
Tsumura-Saireito vs CM	1	RR 1.80 (0.68 to 4.78)	0.24	Sato *et al*^38^
Qingqiao capsule vs CM	1	RR 1.69 (1.04 to 2.75)	0.03	Sun *et al*^34^
Evaluation restoration time of hearing				
Qingqiao capsule vs CM	1	MD −1.80 (−3.26 to −0.34)	0.02	Sun *et al*^34^
Proportion of patients with normalised tympanometry				
Tsumura-Saireito vs CM	1	RR 9.14 (1.18 to 70.61)	0.03	Sato *et al*^38^

CM, conventional medicine; MD, mean difference; RR, risk ratio.

#### Complete or partial resolution

Complete or partial resolution was evaluated by two or more of combined assessment of the presence of symptoms, otoscopy and tympanometry in all studies. All trials^22–38^ evaluated either complete or partial resolution at various time points. Two trials^31^ ^34^ assessed the complete resolution of OME at 2 or 3 months post randomisation, and showed borderline significant improvements with Qingqiao capsule (RR: 2.33, 95% CI 0.98 to 5.53, p=0.05),^34^ but no differences with an unnamed HM (RR: 1.01, 95% CI 0.51 to 2.00, p=0.98)^31^ between the treatment groups.

Two trials^33^ ^36^ estimated the complete resolution rate within 1 week and demonstrated statistically significant improvements with the Huanglong tonger pill (RR: 2.12, 95% CI 1.94 to 2.31, p<0.00001) or Erzhang decoction (RR: 1.65, 95% CI 1.07 to 2.54, p=0.02) plus CM versus CM alone. Of the eight trials^22^ ^23^ ^27^ ^29^ ^31–33^ ^37^ that evaluated effects between 1 and 2 weeks, three^29^ ^33^ ^37^ reported favourable effects with HM plus CM versus CM alone. Compared with CM alone, the Huanglong tonger pill plus CM^33^ ^37^ yielded significant effects after a 10-day treatment course (RR: 1.63, 95% CI 1.55 to 1.73, p<0.00001), and one trial^29^ reported favourable effects of HM plus CM versus CM alone (RR: 3.15, 95% CI 1.37 to 7.26, p=0.007). No significant improvements were observed in the other trials.^22^ ^23^ ^27^ ^31^ ^32^

Of the five trials^25^ ^26^ ^28^ ^30^ ^35^ that evaluated the effects of treatment between 2 and 4 weeks, one^28^ reported that Tongqiao tablets were superior to amoxicillin plus terfenadine (RR: 3.05, 95% CI 1.23 to 7.54, p=0.02), and another trial^30^ reported favourable effects with HM when compared with cephradine plus prednisone plus mucosolvan (RR: 1.43, 95% CI 1.11 to 1.84, p=0.006). Two trials^25^ ^35^ that compared HM plus CM with CM alone reported statistically marginal effects (p=0.05). However, two additional trials that compared HM with CM did not report statistically significant improvements.^25^ ^26^ In one trial^38^ that estimated partial OME resolution after a 4-week treatment course, Tsumura-Saireito was found to have a more favourable effect than cepharanthin (RR: 2.33, 95% CI 1.03 to 5.30, p=0.04). After 6 weeks, a Tongqiao huoxue decoction^24^ was superior to roxithromycin plus prednisolone acetate (RR: 3.35, 95% CI 1.80 to 6.24, p=0.0001).

#### Improvements in hearing

Three trials^22^ ^34^ ^38^ provided data regarding improvements in hearing; of these, two trials^22^ ^38^ measured hearing threshold level differences before and after treatment using pure tone audiometry, and one trial^34^ assessed the hearing restoration rate and elapsed time using pure tone threshold results but did not include detailed criteria. Two additional trials^34^ ^38^ evaluated improvements in clinical hearing symptoms, and one trial^38^ determined that an improvement in pure tone audiometry exceeding 15 dB indicated efficacy.

In three trials,^22^ ^34^ ^38^ the Qingqiao capsule^34^ yielded significant improvements in the restoration rate (RR: 1.61, 95% CI 1.12 to 2.32, p=0.010) and time (MD: −1.70, 95% CI −2.50 to −0.90, p<0.0001) relative to the cefaclor capsule. The Qingqiao capsule also yielded favourable effects on the proportion of patients who achieved hearing improvements (RR: 1.69, 95% CI 1.04 to 2.75, p=0.03) and the assessed clinical hearing restoration time (MD: −1.80, 95% CI −3.26 to −0.34, p=0.02). Compared with CM alone, the Tongqiao huoxue decoction plus CM^26^ significantly reduced the hearing threshold levels (MD: 5.80, 95% CI 2.44 to 9.16, p=0.0007), and Tsumura-Saireito^38^ was found to affect the proportion of patients with normalised tympanometry (RR: 9.14, 95% CI 1.18 to 70.61, p=0.03). In contrast, Tsumura-Saireito^38^ had non-significant effects on differences in the hearing threshold level (MD: 3.30, 95% CI −1.88 to 8.48, p=0.21) and the proportion of patients who achieved hearing improvements (RR: 1.80, 95% CI 0.68 to 4.78, p=0.24). Detailed data for hearing thresholds before and after therapy are presented in online supplementary appendix 4.

10.1136/bmjopen-2016-011250.supp4 supplementary appendix 4

#### Adverse events

Only five of the included RCTs^28–30^ ^34^ ^38^ mentioned adverse events. None of the RCTs reported any serious adverse effects. Three RCTs^28^ ^29^ ^38^ reported no adverse effects during the study period, whereas one RCT^34^ reported that two patients in the control group suffered from nausea, vomiting, diarrhoea and urticaria, with no reported adverse effects in the intervention group. One trial^30^ reported the following: one case of headache and dizziness in the intervention group and two cases of headache and dizziness, two of nausea and vomiting and one of xerostomia in the control group.

#### Other outcomes

We had planned to analyse the effects of treatment on language and speech development, cognitive development, ventilation tube insertion, tympanic membrane sequelae, reductions in OME-associated complications and the quality of life but were unable to determine these results because of a lack of data. Given the inconsistency among the included trials, we were also unable to conduct our planned subgroup and sensitivity analyses.

## Discussion

### Summary of the main results

From the 17 included RCTs, we determined that several HM formulations (Tongqiao tablets, unnamed HM, Tongqiao huoxue decoction, Tsumura-Saireito) appeared to be more effective than CM in terms of the complete or partial resolution rates of clinical symptoms and signs. Similar effects were observed when Huanglong tonger pills, an Erzhang decoction, two unnamed HM and Shenling baizhu powder were combined with CM and compared with CM alone. Other HM prescriptions and combination therapies involving HM and CM did not yield favourable effects when compared with CM alone.

In the three RCTs that assessed hearing symptoms, Qingqiao capsules were associated with a statistically significant improvement in hearing symptoms when compared with CM. Furthermore, a Tongqiao huoxue decoction plus CM appeared to be more effective than CM alone in terms of improved pure tone threshold levels. These results suggest that each HM prescription has a different bioactive effect with respect to OME. Finally, no severe adverse effects were observed in the HM groups, suggesting that HM may be safe for patients with OME.

### Overall completeness and applicability of evidence and implications for clinical practice

Although several HM formulations appeared to be potentially effective against OME, we were unable to draw conclusions regarding the applicability for clinical practice because of the lack of evidence, low quality of the available evidence and heterogeneity of HMs included in this study. By including all types of HMs, we were able to provide overview of the HMs prescribed to OME patients, but we were unable to synthesise the evidence for the use of HM in OME.

Although such limitations do not always mean that the treatment is ineffective, they might indicate that the effectiveness has not been adequately investigated. Larger, more rigorous and adequately powered multicentre randomised clinical evaluations of HM for OME are thus warranted.

### Quality of evidence and potential biases in the review

All trials were methodologically weak and had a high risk of bias. Only one of the 17 trials provided information about the randomisation method and implemented double blinding. As the other 16 trials used different types of CM with different appearances, patient and practitioner blinding might have been impossible.

Although we were unable to find protocols for the included trials, selective reporting bias might have been present, as none of the trials reported losses to follow-up and only five trials reported adverse effects. In addition, the sample size calculation was potentially defective because statistical power could not be guaranteed for the studies. In addition, inadequate information was provided about the quality standards used during HM manufacturing, and therefore the ingredients and their compositions were not standardised. All of these potential biases might have interfered with the true evaluation of these interventions.

### Comparison with other reviews

No previous reviews of oral administration of HM for OME were identified in peer-reviewed journals. However, one review evaluated CM and complementary medicine for the treatment of paediatric otitis media. Levi *et al*^39^ reported that topical HM ear drops might be beneficial, although the efficacy of this treatment was unclear because of variations in the compositions of these drops (usually a combination of marigold (*Calendula flores*), garlic (*Allium sativum*), mullein (*Verbascum thapsus*), St. John's wort (*Hypericum perforatum*), lavender and vitamin E).

### Implications for research

We propose the following suggestions for future research endeavours:

*(1) Development of an adequate placebo:* The unique formulations of HMs are the main cause of inadequate blinding. For example, even though a double-blinded study might administer treatments in the same shape and format for comparative purposes, the treatments might have different flavours, potentially leading to the failure of participant and practitioner blinding. This is a fundamental challenge of clinical trials of HM. The development of placebos identical in shape and flavour to the experimental HM is an important area of further research.

*(2) Development of HM dosage guidelines:* HM dosages varied among the included trials, and of the 15 trials that involved participants younger than 18 years, only two mentioned HM dosage rules for paediatric participants (ie, ‘half dose for those younger than 14 years’ or ‘adjust dosage according to age’). Appropriate HM dosages did not appear to be calculated according to the participants' ages and weights, and this omission might have been because of the lack of appropriate herbal prescription dosage guidelines. Further studies to investigate the adequate dosages of HMs based on pharmacokinetic and pharmacodynamic data are greatly needed.

*(3) Evaluation of drug compliance:* As HMs have distinctive flavours, drug compliance might also have affected the study outcomes. In future, drug compliance should be evaluated.

## Conclusions

There are some indications that several HM formulations could potentially improve the rate of complete sign and symptom (including hearing symptoms) resolution among patients with OME. However, given the low quality of evidence, these results should be interpreted with caution. Further investigation of the effects and safety of HM for patients with OME through rigorously designed randomised trials should be conducted.
