# Supplementary material for: Herbal medicines for the treatment of otitis media with effusion: a systematic review of randomised controlled trials
Source: BMJ Open. 2016 Nov 24;6(11):e011250. doi: 10.1136/bmjopen-2016-011250 (PMC5168523; doi:10.1136/bmjopen-2016-011250)
Supplement: supplementary appendix 1 [file bmjopen-2016-011250supp_appendix1.pdf]

Appendix 1. MEDLINE Search Strategy

#1 otitis media with effusion [MeSH Terms]

#2 ear, middle secretion

#3 glue ear [Title/Abstract]

#4 ((otitis [Title/Abstract]) AND media [Title/Abstract]) AND effusion* [Title/Abstract]

#5 ((middle [Title/Abstract]) AND ear [Title/Abstract]) AND effusion* [Title/Abstract]

#6 nonsuppurative otitis [Title/Abstract]

#7 tympanitis [Title]

#8 serous otitis [Title]

#9 secretory otitis [Title]

#10 otitis serosa [Title]

#11 (mucoid [Title]) AND otitis [Title]

#12 (seromuco* [Title]) AND otitis [Title]

#13 ((sero [Title]) AND muco* [Title]) AND otitis [Title]

#14 ((mucoid [Title]) AND middle [Title]) AND ear [Title]

#15 ((seromuc* [Title]) AND middle [Title]) AND ear [Title]

#16 (adhesive [Title]) AND otitis [Title]

#17 (exudative [Title]) AND otitis [Title]

#18 (mucous [Title]) AND otitis [Title]

#19 #1 OR #2 OR #3 OR #4 OR #5 OR #6 OR #7 OR #8 OR #9 OR #10 OR #11 OR #12 OR #13 OR #14 OR #15 OR #16 OR #17 OR #18

#20 traditional medicine, african [MeSH Terms]

#21 traditional medicine, chinese [MeSH Terms]

#22 traditional medicine, east asia [MeSH Terms]

#23 traditional medicine, korean [MeSH Terms]

#24 traditional medicine, mongolian [MeSH Terms]

#25 traditional medicine, oriental [MeSH Terms]

#26 medicine, kampo [MeSH Terms]

#27 medicine, arabic [MeSH Terms]

#28 medicine, ayurvedic [MeSH Terms]

#29 traditional medicine, tibetan [MeSH Terms]

#30 medicine, herbal [MeSH Terms]

#31 phytotherapy [MeSH Terms]

#32 drugs, chinese herbal [MeSH Terms]

#33 plants, medicinal [MeSH Terms]

#34 plant extracts [MeSH Terms]

#35 ethnobotany [MeSH Terms]

#36 ethnopharmacology [MeSH Terms]

#37 plant* [Title/Abstract]

#38 herb* [Title/Abstract]

#39 #20 OR #21 OR #22 OR #23 OR #24 OR #25 OR #26 OR #27 OR #28 OR #29 OR #30 OR #31 OR #32 OR #33 OR #34 OR #35 OR #36 OR #37 OR #38

#40 #19 AND #3
