# Supplementary material for: Herbal medicines for the treatment of otitis media with effusion: a systematic review of randomised controlled trials
Source: BMJ Open. 2016 Nov 24;6(11):e011250. doi: 10.1136/bmjopen-2016-011250 (PMC5168523; doi:10.1136/bmjopen-2016-011250)
Supplement: supplementary appendix 2 [file bmjopen-2016-011250supp_appendix2.pdf]

Appendix 2. Detailed information about herbal prescriptions used in the included trials

First author (Year)	Herbal Medicinal Preparation Name	Pharmaceutical Active Ingredients	Unit Dose	Prescription Method	Pharmaceutical formulation	Administration Method
Chen (2013)[22]	*Tongqiao huoxue decoction*	*Paeoniae Radix Rubra*<br>*Cnidii Rhizoma*<br>*Persicae Semen*<br>*Jujubae Fructus*<br>*Carthami Flos*<br>*Allii Radix*<br>*Zingiberis Rhizoma Recens*<br>*Moschi Moschiferus*<br>*Cimicifugae Rhizoma*<br>*Bupleuri Radix*	3g<br>3g<br>9g<br>10g<br>9g<br>12g<br>9 g<br>0.15g<br>6 g<br>9 g	Herbal preparation extracted decoction with 250 ml yellow rice wine	decoction	Taking herbal decoction once a day at bedtime
Guo (2004)[23]	*Biyan Qingdu Granule*	*Chrysanthemi lndici Flos*<br>*Xanthii Fructus*<br>*Paridis Rhizoma*<br>*Rubus parvifolius*<br>*Zanthoxylum nitidum*<br>*Prunellae Spica*<br>*Gentianae Radix*<br>*Codonopsis Pilosulae Radix*	Not mentioned	Manufactured by pharmaceutical co mpany	granule	20g each time, Dissolve 20g granule in water, 2 times a day
He (2013)[24]	*Tongqiao huoxue decoction*	*Paeoniae Radix Rubra*<br>*Persicae Semen*<br>*Carthami Flos*<br>*Cnidii Rhizoma*<br>*Allii Radix*<br>*Zingiberis Rhizoma Recens*<br>*Moschi Moschiferus*<br>*Bupleuri Radix*<br>*Acori Graminei Rhizoma*<br>*Cimicifugae Rhizoma*	20g<br>12g<br>12g<br>15g<br>12g<br>12g<br>0.15g<br>10g<br>10g<br>10g	Herbal preparation extracted decoction with water	decoction	Taking herbal decoction 500ml divided into 3, 3 times a day

		Jujubae Fructus	10g			
Hu (2000)[25]	-	*Cyperi Rhizoma*<br>*Cnidii Rhizoma*<br>*Bupleuri Radix*<br>**Pattern/Syndrome of qi stagnation and blood stasis**<br>*Paeoniae Radix Rubra*<br>*Acori Graminei Rhizoma*<br>**spleen qi deficiency pattern/syndrome**<br>*Codonopsis Pilosulae Radix*<br>*Atractylodis Rhizoma Alba*<br>*Cimicifugae Rhizoma*<br>**liver-kidney yin deficiency pattern/syndrome**<br>*Rehmanniae Radix Preparata*<br>*Crataegii Fructus*<br>*Schizandrae Fructus*<br>**kidney yang deficiency pattern/syndrome**<br>*Aconiti Lateralis Radix Praeparata*<br>*Crataegii Fructus*<br>*Alismatis Rhizoma*	6g<br>6g<br>6g<br><br>6g<br>3g<br><br>10g<br>10g<br>6g<br><br>10g<br>10g<br>6g<br><br>5g<br>10g<br>12g	Water extracted decoction	decoction	2 times a day
Jiang (2013)[26]	*Shenling baizhu powder*	*Dolichoris Semen*<br>*Atractylodis Rhizoma Alba*<br>*Poria(Hoelen)*<br>*Glycyrrhizae Radix*<br>*Platycodi Radix*<br>*Nelumbinis Semen*<br>*Ginseng Radix*<br>*Amomi Fructus*<br>*Dioscoreae Rhizoma*<br>*Coicis Semen*	Not mentioned	Manufactured by pharmaceutical co mpany	granule	Each time 9g 3 times a day
Li (2014)[27]	-	*Dioscoreae Rhizoma*<br>*Cnidii Rhizoma*<br>*Bupleuri Radix*<br>*Platycodi Radix*<br>*Plantaginis Semen*	10g<br>10g<br>10g<br>10g<br>10g	Herbal preparation extracted decoction with 500ml water	decoction	Taking 100ml herbal decoction, 2 times a day

		Acori Graminei Rhizoma<br>*Pheretimae Corpus*<br>*Xanthii Fructus*<br>*Glycyrrhizae Radix*<br>*Atractylodis Rhizoma Alba*<br>*Poria(Hoelen)*<br>*Salviae Miltiorrhizae Radix*	10g<br>10g<br>10g<br>5g<br>20g<br>20g<br>15g			
Liao (1998)[28]	*Tongqiao tablet*	*Plantaginis Semen*<br>*Radix Ilicis Pubescentis*<br>*Bupleuri Radix*<br>*Poria(Hoelen)*<br>*Cnidii Rhizoma*<br>*Magnoliae Flos*<br>*Acori Graminei Rhizoma*<br>*Pheretimae Corpus*<br>*Glycyrrhizae Radix*	15g<br>30g<br>10g<br>15g<br>5g<br>10g<br>10g<br>12g<br>5g	Produced by preparation department in hospital.<br>Each tablet weighing 0.5g containing 2.24g of crude drugs	Tablet	4 tablets (2.0g) each time, 3 times a day
Liu (2005)[29]	-	*Astragali Radix*<br>*Saposhnikoviae Radix*<br>*Atractylodis Rhizoma Alba*<br>*Poria(Hoelen)*<br>*Alismatis Rhizoma*<br>*Acori Graminei Rhizoma*<br>*Armeniacae Semen*<br>*Cnidii Rhizoma*<br>*Salviae Miltiorrhizae Radix*<br>*Plantaginis Semen*<br>*Coicis Semen*<br>*Cyperi Rhizoma*<br>*Aurantii Fructus Pericarpium*	30g<br>10g<br>10g<br>10g<br>10g<br>10g<br>10g<br>10g<br>10g<br>10g<br>30g<br>10g<br>6g	Herbal preparation extracted decoction with water	decoction	2 times a day
Liu (2014)[30]	-	**Duration of illness≤15day**<br>*Pinelliae Rhizoma*<br>*Magnoliae Flos*<br>*Lonicerae Flos*<br>*Forsythiae Fructus*	 12g<br>12g<br>9g<br>6g	Herbal preparation extracted decoction with 500ml water	decoction	Taking herbal decoction, 2 times a day

		Taraxaci Herbai	12g			
		Fritillariae Bulbus	6g			
		Acori Graminei Rhizoma	6g			
		Glycyrrhizae Radix	3g			
		**Duration of illness>15day, ≤25d**				
		Magnoliae Flos	12g			
		Bupleuri Radix	6g			
		Cnidii Rhizoma	9g			
		Agastachis Herba	6g			
		Acori Graminei Rhizoma	6g			
		Aucklandiae Radix	6g			
		Alismatis Rhizoma	6g			
		Scutellariae Radix	3g			
		**Duration of illness>25day**				
		Magnoliae Flos	12g			
		Mori Radicis Cortex	6g			
		Alismatis Rhizoma	6g			
		Persicae Semen	3g			
		Carthami Flos	3g			
		Citri Pericarpium	3g			
Lu (2013)[31]	-	*Ginseng Radix*<br>*Dolichoris Semen*<br>*Atractylodis Rhizoma Alba*<br>*Poria(Hoelen)*<br>*Coicis Semen*<br>*Platycodi Radix*<br>*Cinnamomi Ramulus*<br>*Acori Graminei Rhizoma*<br>*Liquidambar formosana Hance*<br>*Astragali Radix*	10g<br>15g<br>15g<br>15g<br>20g<br>10g<br>10g<br>10g<br>10g<br>20g	Herbal preparation extracted decoction with 500ml water	decoction	Taking herbal decoction 300ml divided into 2, 2 times a day after the meal
Qu (2013)[32]	-	*Astragali Radix*<br>*Gardeniae Fructus*<br>*Codonopsis Pilosulae Radix*<br>*Rehmanniae Radix*	18g<br>18g<br>18g<br>12g	Herbal preparation extracted decoction with water	decoction	Taking 150ml herbal decoction, 3 times a day

		Puerariae Radix<br>*Cimicifugae Rhizoma*<br>*Phellodendri Cortex*<br>*Paeoniae Radix Alba*<br>*Viticis Fructus*<br>*Gentianae Radix*<br>*Glycyrrhizae Radix*	30g<br>10g<br>15g<br>15g<br>15g<br>15g<br>6g			
Sato (1988)[38]	*Tsumura-Saireito*	*Alismatis Rhizoma*<br>*Atractylodis Rhizoma Alba*<br>*Bupleuri Radix*<br>*Cinnamomi Cortex Spissus*<br>*Zingiberis Rhizoma Recens*<br>*Ginseng Radix*<br>*Zizyphi Fructus*<br>*Glycyrrhizae Radix*<br>*Pinelliae Rhizoma*<br>*Polyporus*<br>*Poria(Hoelen)*<br>*Scutellariae Radix*	Not mentioned	Manufactured by pharmaceutical co mpany	granule	1.5g each time, 2 time a day
Shi (2005)[33]	*Huanglong tonger pill*	*Gentianae Radix*<br>*Scutellariae Radix*<br>*Alismatis Rhizoma*<br>*Magnoliae Cortex*<br>*Gardeniae Fructus*<br>*Akebiae Caulis*<br>*Plantaginis Semen*<br>*Acori Graminei Rhizoma*<br>*Atractylodis Rhizoma Alba*<br>*Polyporus*<br>*Bupleuri Radix*<br>*Coptidis Rhizoma*<br>*Poria(Hoelen)*	12g<br>10g<br>10g<br>10g<br>10g<br>10g<br>12g<br>12g<br>12g<br>12g<br>9g<br>5g<br>15g	2 mm diameter pills with Powdered herbal preparation and water	pill	10g each time with warm water, 2 times a day
Sun (2005)[34]	*Qingqiao Capsule*	*Scrophulariae Radix*<br>*Rehmanniae Radix*	Not mentioned	Produced by preparation	capsule	5 capsules each time, 3 times a

		Salviae Miltiorrhizae Radix<br>*Achyranthis Radix*<br>*Bombycis Corpus cum Batryticatus*<br>*Angelicae Sinensis Radix*<br>*Coicis Semen*<br>*Amomi Rotundus Fructus*<br>*Glycyrrhizae Radix*<br>*3 more unknown herbal preparation*		department in hospital.<br>Each capsule weighing 0.45g containing 2.25g of crude drugs		day
Tian (2014)[35]	*Shenling baizhu powder*	*Dolichoris Semen*<br>*Atractylodis Rhizoma Alba*<br>*Poria(Hoelen)*<br>*Platycodi Radix*<br>*Nelumbinis Semen*<br>*Ginseng Radix*<br>*Amomi Fructus*<br>*Dioscoreae Rhizoma*<br>*Coicis Semen*<br>*Glycyrrhizae Radix*	Not mentioned	Manufactured by pharmaceutical co mpany	granule	Each time 9g, 3 times a day
Zhang (2013)[36]	*Erzhang decoction*	*Lonicerae Flos*<br>*Forsythiae Fructus*<br>*Platycodi Radix*<br>*Acori Graminei Rhizoma*<br>*Citri Pericarpium*<br>*Poria(Hoelen)*<br>*Sojae Semen Praeparatum*<br>*Mentha arvensis var. piperascens Makinv.*<br>*Schizonepetae Herba*<br>*Cnidii Rhizoma*<br>*Bupleuri Radix*<br>*Glycyrrhizae Radix Praeparata*	10g<br>12g<br>10g<br>10g<br>10g<br>12g<br>9g<br>9g<br>10g<br>10g<br>10g<br>3g	Herbal preparation extracted decoction with water	decoction	Taking 150ml herbal decoction, 3 times a day after the meal
Zhao (2012)[37]	*Huanglong tonger pill*	*Polyporus*<br>*Atractylodis Rhizoma Alba*<br>*Acori Graminei Rhizoma*	12g<br>12g<br>12g	2 mm diameter pills with Powdered herbal preparation	pill	10g each time with warm water, 2 times a

Gentianae Radix	12g	and water	day
Plantaginis Semen	12g		
Akebiae Caulis	10g		
Gardeniae Fructus	10g		
Magnoliae Cortex	10g		
Alismatis Rhizoma	10g		
Scutellariae Radix	10g		
Poria(Hoelen)	15g		
Coptidis Rhizoma	5g		
Bupleuri Radix	9g		
