# Supplementary material for: Herbal medicines for the treatment of otitis media with effusion: a systematic review of randomised controlled trials
Source: BMJ Open. 2016 Nov 24;6(11):e011250. doi: 10.1136/bmjopen-2016-011250 (PMC5168523; doi:10.1136/bmjopen-2016-011250)
Supplement: supplementary appendix 3 [file bmjopen-2016-011250supp_appendix3.pdf]

Appendix 3. Websites presenting herbal remedies and single herbs pictures

First author (Year)	Herbal Medicinal Preparation Name	Picture(Link)
Guo(2004)	Biyan Qingdu Granule	https://www.acuatlanta.net/star-ring-bi-yan-qing-du-chong-ji-10-packets-p-66175.html?mclick=tags&osCsid=1c1524c978696b21765eb577ac4d0110
Jiang(2013) Tian(2014)	Shenling baizhu powder	https://www.goldenneedleonline.com/Shen-Ling-Bai-Zhu-San-Granules-EF290.html
Sato(1988)	Tsumura-Saireito	https://www.mimaki-family-japan.com/item/detail?item_prefix=TF&item_code=005338&item_branch=002

Pharmaceutical Active Ingredients	Picture(Link)
Achyranthis Radix	http://old.tcmwiki.com/wiki/radix-achyranthis-bidentatae
Acori Graminei Rhizoma	http://old.tcmwiki.com/wiki/rhizoma-acori-graminei
Agastachis Herba	http://old.tcmwiki.com/wiki/%E8%97%BF%E9%A6%99
Akebiae Caulis	http://old.tcmwiki.com/wiki/caulis-akebiae
Alismatis Rhizoma	http://old.tcmwiki.com/wiki/rhizoma-alismatis
Allii Radix	http://old.tcmwiki.com/wiki/radix-allii-fistulosi-fibrosa
Amomi Fructus	http://old.tcmwiki.com/wiki/%E7%A0%82%E4%BB%81
Amomi Rotundus Fructus	http://old.tcmwiki.com/wiki/fructus-amomi-rotundus
Angelicae Sinensis Radix	http://old.tcmwiki.com/wiki/radix-angelicae-sinensis
Armeniacae Semen	http://old.tcmwiki.com/wiki/semen-armeniacae-amarae
Astragali Radix	http://old.tcmwiki.com/wiki/radix-astragali
Atractylodis Rhizoma Alba	http://old.tcmwiki.com/wiki/bai-zhu
Aucklandiae Radix	http://old.tcmwiki.com/wiki/radix-aucklandiae
Aurantii Fructus Pericarpium	http://old.tcmwiki.com/wiki/%E6%9E%B3%E5%A3%B3
Bombycis Corpus cum Batryticatus	http://old.tcmwiki.com/wiki/%E5%83%B5%E8%A0%B6
Bupleuri Radix	http://old.tcmwiki.com/wiki/radix-bupleuri
Carthami Flos	http://old.tcmwiki.com/wiki/flos-carthami
Chrysanthemi lndici Flos	http://old.tcmwiki.com/wiki/flos-chrysanthemi-indici
Cimicifugae Rhizoma	http://old.tcmwiki.com/wiki/rhizoma-cimicifugae
Cinnamomi Cortex Spissus	http://old.tcmwiki.com/wiki/cortex-cinnamomi
Cinnamomi Ramulus	http://old.tcmwiki.com/wiki/ramulus-cinnamomi
Citri Pericarpium	http://old.tcmwiki.com/wiki/pericarpium-citri-reticulatae
Cnidii Rhizoma	http://old.tcmwiki.com/wiki/%E5%B7%9D%E8%8A%8E
Codonopsis Pilosulae Radix	http://old.tcmwiki.com/wiki/dang-shen
Coicis Semen	http://old.tcmwiki.com/wiki/semen-coicis
Coptidis Rhizoma	http://old.tcmwiki.com/wiki/rhizoma-coptidis
Crataegi Fructus	http://old.tcmwiki.com/wiki/%E5%B1%B1%E6%A5%82

Cyperi Rhizoma	http://old.tcmwiki.com/wiki/rhizoma-cyperi
Dioscoreae Rhizoma	http://old.tcmwiki.com/wiki/%E5%B1%B1%E8%97%A5
Dolichoris Semen	http://old.tcmwiki.com/wiki/%E7%99%BD%E6%89%81%E8%B1%86
Forsythiae Fructus	http://old.tcmwiki.com/wiki/fructus-forsythiae
Fritillariae Bulbus	http://old.tcmwiki.com/wiki/%E8%B2%9D%E6%AF%8D
Gardeniae Fructus	http://old.tcmwiki.com/wiki/fructus-gardeniae
Gentianae Radix	http://old.tcmwiki.com/wiki/radix-gentianae
Ginseng Radix	http://old.tcmwiki.com/wiki/ren-shen
Glycyrrhizae Radix	http://old.tcmwiki.com/wiki/radix-glycyrrhizae
Glycyrrhizae Radix Praeparata	http://old.tcmwiki.com/wiki/radix-glycyrrhizae
Jujubae Fructus	http://old.tcmwiki.com/wiki/fructus-jujubae
Liquidambar formosana Hance	http://old.tcmwiki.com/wiki/%E8%98%87%E5%90%88%E9%A6%99
Lonicerae Flos	http://old.tcmwiki.com/wiki/flos-lonicerae
Magnoliae Cortex	http://old.tcmwiki.com/wiki/cortex-magnoliae-officinalis
Magnoliae Flos	http://old.tcmwiki.com/wiki/xin-yi
Mentha arvensis var. piperascens Makinv.	http://old.tcmwiki.com/wiki/%E8%96%84%E8%8D%B7
Mori Radicis Cortex	http://old.tcmwiki.com/wiki/%E6%A1%91%E7%99%BD%E7%9A%AE
Moschiferus Moschus	http://old.tcmwiki.com/wiki/moschus
Nelumbinis Semen	http://old.tcmwiki.com/wiki/semen-nelumbinis
Paeoniae Radix Alba	http://old.tcmwiki.com/wiki/radix-paeoniae-alba
Paeoniae Radix Rubra	http://old.tcmwiki.com/wiki/radix-paeoniae-rubra
Paridis Rhizoma	http://old.tcmwiki.com/wiki/rhizoma-paridis
Persicae Semen	http://old.tcmwiki.com/wiki/semen-persicae
Saposhnikoviae Radix	http://old.tcmwiki.com/wiki/%E9%98%B2%E9%A2%A8
Phellodendri Cortex	http://old.tcmwiki.com/wiki/cortex-phellodendri
Pheretimae Corpus	http://old.tcmwiki.com/wiki/%E8%9A%AF%E8%9A%93%E6%B3%A5
Pinelliae Rhizoma	http://old.tcmwiki.com/wiki/rhizoma-pinelliae
Plantaginis Semen	http://old.tcmwiki.com/wiki/semen-plantaginis
Platycodi Radix	http://old.tcmwiki.com/wiki/radix-platycodi
Polyporus	http://old.tcmwiki.com/wiki/polyporus
Poria(Hoelen)	http://old.tcmwiki.com/wiki/poria
Prunellae Spica	http://old.tcmwiki.com/wiki/spica-prunellae
Puerariae Radix	http://old.tcmwiki.com/wiki/radix-puerariae
Aconiti Lateralis Radix Praeparata	http://old.tcmwiki.com/wiki/radix-aconiti-lateralis-praeparata
Radix Ilicis Pubescentis	http://old.tcmwiki.com/wiki/radix-ilicis-pubescentis
Rehmanniae Radix	http://old.tcmwiki.com/wiki/radix-rehmanniae
Rehmanniae Radix Preparata	http://old.tcmwiki.com/wiki/%E7%86%9F%E5%9C%B0%E9%BB%83
Rubus parvifolius	http://old.tcmwiki.com/wiki/%E8%A6%86%E7%9B%86%E5

	%AD%90
Salviae Miltiorrhizae Radix	http://old.tcmwiki.com/wiki/radix-salviae-miltiorrhizae
Schizandrae Fructus	http://old.tcmwiki.com/wiki/fructus-schisandrae
Schizonepetae Herbal	http://old.tcmwiki.com/wiki/herba-schizonepetae
Scrophulariae Radix	http://old.tcmwiki.com/wiki/radix-scrophulariae
Scutellariae Radix	http://old.tcmwiki.com/wiki/radix-scutellariae
Sojae Semen Praeparatum	http://old.tcmwiki.com/wiki/semen-sojae-preparatum
Taraxaci Herba	http://old.tcmwiki.com/wiki/herba-taraxaci
Viticis Fructus	http://old.tcmwiki.com/wiki/fructus-viticis
Xanthii Fructus	http://old.tcmwiki.com/wiki/fructus-xanthii
Zingiberis Rhizoma Recens	http://old.tcmwiki.com/wiki/rhizoma-zingiberis-recens
Zizyphi Fructus	http://old.tcmwiki.com/wiki/%E5%A4%A7%E6%A3%97
