# Supplementary material for: Herbal medicines for the treatment of otitis media with effusion: a systematic review of randomised controlled trials
Source: BMJ Open. 2016 Nov 24;6(11):e011250. doi: 10.1136/bmjopen-2016-011250 (PMC5168523; doi:10.1136/bmjopen-2016-011250)
Supplement: supplementary appendix 4 [file bmjopen-2016-011250supp_appendix4.pdf]

Appendix 4. Details of hearing threshold levels of included trials

Chen(2013): hearing threshold level differences

	Pre-treatment(dB)	Post-tre;atment(dB)	Differences(dB)
**Treatment group**	47.4±6.52	25.2±9.94	20.1±8.40
**Control group**	45.8±7.60	30.7±10.52	14.3±7.13

Sato(1988): hearing threshold level differences

	Pre-treatment(dB)	Post-treatment(dB)	Differences(dB)
**Treatment group**	30.1±6.7	22.9±8.6	7.1±8.9
**Control group**	31.9±8.3	28.1±11.6	3.8±12.0

Sun(2005): Elapsed time that the hearing threshold levels reach under the 15 dB

	Treatment group	Control group
**Elapsed time**	12.6±2.3	14.3±0.4
